# MiPepid: MicroPeptide identification tool using machine learning

**DOI:** 10.1186/s12859-019-3033-9

**Published:** 2019-11-08

**Authors:** Mengmeng Zhu, Michael Gribskov

**Affiliations:** 1 Department of Statistics, Purdue University, West Lafayette, IN 47907 USA; 2 Department of Biological Sciences, Purdue University, West Lafayette, IN 47907 USA

**Keywords:** Micropeptide, Small ORF, sORF, smORF, Coding, Noncoding, lncRNA, Machine learning

## Abstract

**Background:**

Micropeptides are small proteins with length < = 100 amino acids. Short open reading frames that could produces micropeptides were traditionally ignored due to technical difficulties, as few small peptides had been experimentally confirmed. In the past decade, a growing number of micropeptides have been shown to play significant roles in vital biological activities. Despite the increased amount of data, we still lack bioinformatics tools for specifically identifying micropeptides from DNA sequences. Indeed, most existing tools for classifying coding and noncoding ORFs were built on datasets in which “normal-sized” proteins were considered to be positives and short ORFs were generally considered to be noncoding. Since the functional and biophysical constraints on small peptides are likely to be different from those on “normal” proteins, methods for predicting short translated ORFs must be trained independently from those for longer proteins.

**Results:**

In this study, we have developed MiPepid, a machine-learning tool specifically for the identification of micropeptides. We trained MiPepid using carefully cleaned data from existing databases and used logistic regression with 4-mer features. With only the sequence information of an ORF, MiPepid is able to predict whether it encodes a micropeptide with 96% accuracy on a blind dataset of high-confidence micropeptides, and to correctly classify newly discovered micropeptides not included in either the training or the blind test data. Compared with state-of-the-art coding potential prediction methods, MiPepid performs exceptionally well, as other methods incorrectly classify most bona fide micropeptides as noncoding. MiPepid is alignment-free and runs sufficiently fast for genome-scale analyses. It is easy to use and is available at https://github.com/MindAI/MiPepid.

**Conclusions:**

MiPepid was developed to specifically predict micropeptides, a category of proteins with increasing significance, from DNA sequences. It shows evident advantages over existing coding potential prediction methods on micropeptide identification. It is ready to use and runs fast.

**Electronic supplementary material:**

The online version of this article (10.1186/s12859-019-3033-9) contains supplementary material, which is available to authorized users.

## Background

Micropeptides are generally defined as small proteins of <= 100 amino acid residues [1–3]. Their existence was traditionally ignored because few micropeptides had been shown to be functionally important, mostly due to technological limitations in isolating small proteins [4]. Consequently, small open reading frames (sORFs or smORFs, <= 303 bp) that encode micropeptides are generally ignored in gene annotation and have been considered to be noise (occurring by chance) and to be unlikely to be translated into proteins [2, 4, 5].

With improved technology, an increasing number of micropeptides have been discovered, and have been shown to play important roles in muscle performance [6], calcium signaling [7], heart contraction [8], insulin regulation [9], immune surveillance [10, 11], etc. In particular, many micropeptides were shown to be translated from transcripts that were previously annotated as putative long noncoding RNAs (lncRNAs) [12, 13]. This fact challenges the “noncoding” definition and raises questions about the functional mechanisms of lncRNAs, i.e., whether they function through their 3D RNA structure, or via the micropeptides translated from encoded sORFs, or both.

With the increasing recognition of the importance of the “once well forgotten” field of micropeptides, it is increasingly important to develop a large-scale method for identifying them in a cost-effective way. Ribosome profiling [14, 15] (Ribo-Seq) is a recent high-throughput technique for identifying potentially coding sORFs by sequencing mRNA fragments captured with translating ribosomes. Despite its advantages, there currently is no community consensus on how Ribo-Seq data should be used for gene annotation [16], as some investigators have questioned whether capture of RNAs by ribosomes necessarily implies translation; some capture could be transient or non-specific rather than truly functional [17, 18]. Ribo-Seq requires the use of next generation sequencing and thus has significant costs. In addition, depending on the sequencing depth and quality, it may suffer from false positives, and may not reveal all coding sORFs due to differences in sORF expression in different tissues, developmental stages, and conditions. Therefore, the sORFs discovered from Ribo-Seq still require experimental verification of their coding potentials.

It is much less expensive to predict coding sORFs from DNA sequences using bioinformatic tools. Although experimental verification is still required for predicted sORFs, a bioinformatic prediction of the coding potential of any sORFs before experimental verification is valuable since bioinformatics analysis costs almost nothing and could potentially provide useful insights.

There are currently few bioinformatic tools specifically designed for predicting the coding potential of small ORFs. uPEPperoni [19] is a web server designed to detect sORFs in the 5′ untranslated regions (5′-UTR) of mRNAs. It detects conserved sORFs without explicitly predicting their coding potential. Although 5′-UTR sORFs are an important component of the sORF population, many sORFs are located elsewhere, such as within the coding region of an mRNA, in lncRNAs, etc. The sORF finder [20] program specifically identifies sORFs using the nucleotide frequency conditional probabilities of the sequence, however it was developed nearly a decade ago, and the server is no longer accessible. In addition, because many micropeptides have been discovered in the last decade, a much larger training dataset can now be assembled, and this should greatly improve the prediction quality. Data pipelines have been described [21–23] that calculate the coding abilities of sORFs, especially those identified from Ribo-Seq data; however, these pipelines are not standalone packages readily available for other users. Other well-known coding potential prediction tools such as CPC [24], CPC2 [25], CPAT [26], CNCI [27], PhyloCSF [28], etc. which were trained on datasets consisting primarily of normal-sized proteins. Because of the differences between sORF peptides and globular proteins, and because these methods were not trained on large sORF datasets, it is likely they do not perform well in sORF prediction (as shown in the Results section below). In general, most coding potential predictors penalize short ORFs and those that lack significant similarity to known proteins; both of these factors compromise the ability of existing tools to correctly predict sORFs.

With the ongoing development of techniques such as Ribo-Seq and mass spectrometry (MS), an increasing number of micropeptides have been experimentally identified and verified. We have a reasonable amount of data that can be leveraged for the development of bioinformatics tools specifically for micropeptide prediction. sORFs.org [4, 5] is a repository of small ORFs identified specifically from Ribo-Seq and MS data. And SmProt [29] is a database of micropeptides collected from literature mining, known databases, ribosome profiling, and MS.

Machine learning (ML) is a set of algorithms for learning hidden patterns within a set of data in order to classify, cluster, etc. The development of a successful ML-based method for a particular problem depends on a good dataset (clean, with sufficient data, etc.), and a good choice of specific ML algorithm. ML has been used in developing numerous bioinformatics tools, and has been used, for instance, in ORF coding potential prediction [24–27].

In this study, we present MiPepid, a ML-based tool specifically for identifying micropeptides directly from DNA sequences. It was trained using the well-studied logistic regression model on a high-quality dataset, which was carefully collected and cleaned by ourselves. MiPepid achieves impressive performance on several blind test datasets. Compared with several existing state-of-the-art coding potential prediction tools, MiPepid performs exceptionally well on bona fide micropeptide datasets, indicating its superiority in identifying small-sized proteins. It is also a lightweight and alignment-free method that runs sufficiently fast for genome-scale analyses and scales well.

## Implementation

### Datasets

To collect positive as well as negative datasets for micropeptides that are representative yet concise, we selected 2 data sources: SmProt [29] and traditional noncoding RNAs.

### The positive dataset

SmProt [29] is a database of small proteins / micropeptides which includes data from literature mining, known databases (UniProt [30], NCBI CCDS [31–33]), Ribo-Seq, and MS. In particular, SmProt contains a high-confidence dataset consisting of micropeptide data that were collected from low-throughput literature mining, known databases, and high-throughput literature mining data or Ribo-Seq data with supporting MS evidence.

The SmProt high-confidence dataset (containing 12,602 human micropeptides in total) is a reliable data source for positive data since many of the peptides have been experimentally verified, and the rest are supported by multiple evidence. Based on this dataset, we cleaned our own positive dataset using the following pipeline:

1. Obtain the nucleotide sequences of the data. In SmProt, only the amino acid sequences rather than the DNA sequences are provided, although for the majority of data points their corresponding transcript IDs (primarily in Ensembl [34], with others in RefSeq [34] or NONCODE [34]) are provided. Since the DNA sequence of a micropeptide contains essential information that the translated sequence cannot provide (such as nucleotide frequency, etc.), we therefore obtained the corresponding DNA sequences by mapping the protein sequences back to their corresponding transcripts using GeneWise [34]. To ensure the quality of the dataset, only micropeptides that gave a perfect match (no substitutions or indels) were retained.
2. Obtain a nonredundant positive dataset. Proteins with similar sequences may share similar functions, and families of related sequences create a bias towards certain sequence features. To ensure that our positive dataset is not biased by subgroups of micropeptides with similar sequences, we selected a nonredundant set with protein sequence identity ≤0.6. This serves as our **positive** dataset and it contains 4017 data points.

### The negative dataset

It is hard to define a truly negative dataset for micropeptides as more and more sequences that were formerly considered noncoding have been shown to encode translated proteins, such as 5′-UTRs of mRNAs, lncRNAs, etc. Despite the limitations of our current knowledge, we are still able to collect ORFs that are highly likely to be noncoding.

Traditional noncoding RNAs, such as microRNA (miRNA), ribosomal RNA (rRNA), small nuclear RNA (snRNA), etc. are highly likely to be truly noncoding. While there is growing evidence that lncRNAs [35, 36] may sometimes encode translated sORFs, the possibility of sORFs in traditional noncoding RNAs has seldom been mentioned or discussed in literature. In addition, some pipelines for predicting coding regions from Ribo-Seq data utilized those ncRNAs to construct their negative datasets [21, 37]. While there are examples of lncRNAs and “noncoding” regions of mRNAs that encode micropeptides in the SmProt high-confidence dataset, there are no examples of micropeptides encoded by traditional ncRNAs.

We therefore chose human miRNA, rRNA, snRNA, snoRNA (small nucleolar RNA), tRNA (transfer RNA), and scaRNA (small Cajal body RNA, a nucleolar RNA) as the data source for our negative dataset. We selected all human transcripts in the Ensembl database [34] annotated with these 6 biotypes and extracted all possible ORFs from those transcripts, i.e., ORFs with valid start and stop codons from all 3 translation frames. Although there is evidence that non-ATG codons sometimes serve as sORF start codons [5], to ensure the validity of our dataset, we consider only ATG start codons in constructing the negative dataset; in the positive dataset, nearly 99% of sORFs begin with ATG start codons.

We finally gathered 5616 negative sORFs. In the same way as for the positive data, we selected a nonredundant **negative** dataset of size 2936 with pairwise predicted protein sequence identity ≤0.6.

### The training set and the blind test set

We randomly selected 80% of the examples in the positive and negative datasets to build our training set for the machine learning model training; the remaining 20% were used as a blind test set which was only used for model evaluation (Table 1).

**Table 1:** Training and test data sets

Dataset	#Positive	#Negative	#Total
Training	3194	2369	5563
Test	823	567	1390

#positive: number of positive data points

#negative: number of negative data points

#total: total number of data points

### The synthetic_negative dataset

To further test the performance of our method, we generated a synthetic dataset that preserves the length distribution as well as the dinucleotide frequencies [38] of the negative dataset. Since this dataset mimics the negative data, our method is expected to predict negative on this dataset. This **synthetic_negative** dataset is of the same size as the negative dataset (2936), and it was generated using the ushuffle software [39] in the MEME suite [40].

## Methods

### Feature generation

In machine learning, identifying a set of relevant features is the next important step toward constructing a classifier. A set of well-chosen features greatly facilitates differentiating between different classes.

In our study, we believe the key to determining whether a small ORF is translated lies in the nucleotide patterns in the sequence. A translated sORF should have a DNA sequence that is constrained by the physicochemical properties of the translated peptide, the preference of ribosome occupancy, the codon bias of the organism, etc.

*k*-mer features have been widely used to effectively capture nucleotide patterns. A *k*-mer is a subsequence of length *k*, where *k* is an integer ranging from 1 to as high as hundreds depending on the requirements of specific questions. For DNA *k*-mers, there are only 4 types of nucleotides (A, T, C, and G), so the number of distinct *k*-mers for a specific *k* is 4^*k*^. The *k*-mer features are simply encoded as a vector of size 4^*k*^ (denoted as ***v***), with each value in the vector denoting the frequency of one unique *k*-mer in the sequence. If we slide a window of length *k* across the sequence from beginning to end with a step size of *s*, we obtain $\left\lfloor \frac{\left|S\right|-k+1}{s}\right\rfloor$ *k*-mers in total, where ∣ *S*∣ denotes the length of the sequence. Therefore, ${\left|\boldsymbol{v}\right|}_1=\left\lfloor \frac{\left|S\right|-k+1}{s}\right\rfloor$, where |***v***|_1_ is the *L*_1_ norm of ***v***. To exclude the sequence length effects in ***v***, we can use the normalized *k*-mer features, i.e., the *fractional* frequency of each *k*-mer rather than the frequency itself. In this case, |***v***|_1_ = 1.

Regarding the choice of *k*, a hexamer (i.e.*,* 6-mer) is often used in bioinformatics tools for various biological questions [20, 41]. Yet hexamers would give a feature vector of size 4^6^ = 4,096. Compared to 5,563, the size of our training data, a model with as many as 4,096 parameters could potentially overfit the dataset although 5,563 is larger than 4,096. To ensure the generalizability as well as the efficiency of our method, we chose to use 4-mer features. A 4-mer, while short, still captures information about codons, and any dependencies between adjacent amino acid residues since every 4-mer covers parts of 2 adjacent codons / amino acids. A 4-mer feature vector has a reasonable size of 256, much less than 4096, therefore should produce less model overfitting and have shorter running time. To eliminate the length information of a sORF, we chose to use normalized k-mer features. And to better capture the codon information of the translation frame, we chose a step size of 3 for k-mer extraction.

### Logistic regression

From many possible supervised machine learning algorithms, we chose logistic regression for our study. Logistic regression is well-studied and provides easy-to-interpret models that have been shown to be successful in numerous cases and scenarios. The model can be tuned to minimize overfitting by, for instance, including regularization penalties. When used for prediction, the model returns the probability of an instance being in the positive category rather than just a label, which gives more insight into the prediction.

The loss function for logistic regression is:

$$ \underset{\boldsymbol{w},b}{\min}\sum \limits_{i=1}^n\log \left(1+{e}^{-\Big({y}_i\left({X_i}^T\boldsymbol{w}+b\right)}\right)+\lambda {\boldsymbol{w}}^T\boldsymbol{w} $$

, where {*X*_1_, …, *X*_*n*_} are the set of the data points and for each *X*_*i*_ ​, *y*_*i*_ ∈ {−1, +1} is the label. ***w*** is the weight vector and *b* is the bias term. $\sum \limits_{i=1}^n\log \left(1+{e}^{-\Big({y}_i\left({X_i}^T\boldsymbol{w}+b\right)}\right)$ is the negative log likelihood. *λ****w***^*T*^***w*** is the regularization term which helps constrain the parameter space of ***w*** to reduce overfitting, and *λ* is a hyperparameter controlling the regularization strength. For a set of ***w*** and *b*, the classifier assigns the label to data point *X*_*i*_ ​based on the following:

$$ f\left({X}_i\right)=\frac{1}{1+{e}^{-\left({\mathbf{w}}^T{X}_i+b\right)}}\left\{\begin{array}{c}\ge t,{\hat{y}}_i=+1\\ {}<t,{\hat{y}}_i=-1\ \end{array}\right.{\displaystyle \begin{array}{cc}& \\ {}& \end{array}} $$

, where ${\hat{y}}_i$ is the predicted label from the classifier and *t* is the threshold between the positive (+1) and the negative (−1) classes. Although *t* = 0.5 is generally used, 0 ≤ *t* ≤ 1 is also a tunable hyperparameter.

### Performance evaluation

To evaluate the performance of MiPepid and existing methods, we used the following metrics.

1. accuracy

For a dataset *S*, denote the number of correctly classified cases by a method as *c*, then the accuracy is $\frac{c}{\mid S\mid }$, where ∣*S*∣ is the size of the dataset. This definition applies to any dataset used in this paper.

(2) *F*_1_ score

For a dataset that contains both positive and negative data, the *F*_1_ score of the performance of a method on this dataset is:

$$ {F}_1=2\frac{pr\times rc}{pr+ rc} $$

, where *pr* is the precision and *rc* is the recall, and

$$ pr=\frac{TP}{TP+ FP}, rc=\frac{TP}{TP+ FN} $$

, where *TP* is the number of true positives, i.e., the number of correctly classified cases in the positive subset; *FP* is the number of false positives, i.e., the number of misclassified cases in the negative subset; *FN* is the number of false negatives, i.e., the number of misclassified cases in the positive subset. The *F*_1_ score ranges from 0 to 1, with a higher value implying better performance. In this study, the *F*_1_ score is used for the training and the test sets, as both of them consist of both positive and negative data.

### 10-fold cross validation

*N*-fold cross validation is commonly used to select good hyperparameters. Here *n* is an integer ranging from 2 to as high as dozens. In cross validation, the dataset is randomly and evenly divided into *n* folds. For every set of hyperparameter candidates, and for each fold, a model is trained using the other *n* − 1 fold(s) and is evaluated on the left-out fold. The (weighted) average of the *n* evaluations is taken as the overall evaluation for that set of hyperparameter candidates. This cross validation is done for every set of hyperparameter candidates in order to select a set that gives the best performance.

As stated in 3.3, there are 2 hyperparameters in logistic regression: the regularization strength *λ* and the threshold *t*. We performed 10-fold cross validation to tune these 2 hyperparameters. For $\lambda \in \left\{1\mathsf{E}-5,1\mathsf{E}-4,1\mathsf{E}-3,\dots, 1\mathsf{E}+5,1\mathsf{E}+6\right\}$ and *t* ∈ {0, 0.05, 0.1, …, 0.95, 1.0}, we selected the combination of *λ* and *t* that gave the best performance.

### Hyperparameters tuning using 10-fold cross validation

**Fig. 1:**
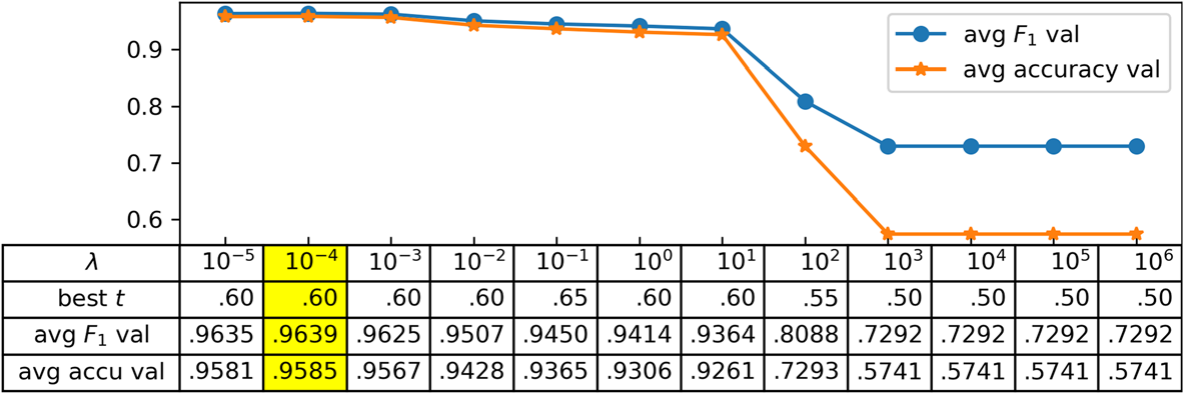
Parameter Optimization. The avg. F_1_ and accuracy are shown at the best *t* for the indicated values of λ. 10-fold cross validation results with different 휆 and 푡 combinations on the training set. *λ*: the hyperparameter for regularization strength in logistic regression; *t*: the hyperparameter for threshold in logistic regression; best *t*: when *λ* is fixed, the *t* from *t* ∈ {0, 0.05, 0.1, …, 0.95, 1.0} that gives the best performance; avg. *F*_1_ val: the average *F*_1_ score on the 10 validation sets when both *λ* and *t* are fixed; avg. accu val: the average accuracy on the 10 validation sets when both *λ* and *t* are fixed

As stated above, in the logistic regression model, the regularization strength *λ* and the threshold *t* are tunable hyperparameters. Therefore, before training the model on the training dataset, we first determined the best combination of *λ* and *t* using 10-fold cross validation. As shown in Fig. 1, when *λ* = 10^−4^ and *t* = 0.60, both the average *F*_1_ (0.9639) and accuracy (0.9585) on the 10 validation sets are the highest.

### Training using the tuned hyperparameters

We therefore chose *λ* = 10^−4^ and *t* = 0.60, and re-trained on the complete training dataset to obtain the MiPepid model. This model achieved an *F*_1_ score of 0.9845 and an overall accuracy of 0.9822 on the training set (Table 2).

**Table 2:** MiPepid results on the training set

*F*_1_	Accuracy		
	Positive	Negative	Overall
0.9845	0.9818	0.9827	0.9822

“positive” and “negative” refer to the accuracies of MiPepid on the positive and negative subsets, respectively;

“overall” refers to the accuracy on the whole training set (positive + negative)

## Results

### MiPepid generalizes well on the hold-out blind test set

The blind test set contains 1390 sequences and was not used during the training stage. As shown in table 3, MiPepid achieved an *F*_1_ score of 0.9640 and an overall accuracy of 0.9576 on this test set. Compared with table 2, although the results are slightly lower, they are still comparably good. In addition, MiPepid performed almost equally well on the positive and negative subsets of the test set as indicated by the corresponding accuracies (0.9587 vs. 09559). Therefore, MiPepid generalizes well and has a balanced performance on both positive and negative data.

**Table 3:** MiPepid results on the blind test set

*F*_1_	Accuracy		
	Positive	Negative	Overall
0.9640	0.9587	0.9559	0.9576

“positive” and “negative” refer to the accuracies of MiPepid on the positive and negative subsets, respectively;

“overall” refers to the accuracy on the whole test set (positive + negative)

### MiPepid performs well on the synthetic_negative dataset

The synthetic_negative dataset mimics the negative dataset by preserving the dinucleotide frequency as well as the length distribution of the real negative data, but because it has been randomized, should have no true sORFs. MiPepid achieved an accuracy of 0.9659 on the synthetic_negative dataset, a very close result to the one on the negative subset of either the training or test set, indicating the robustness of MiPepid.

### MiPepid correctly classifies newly published micropeptides

In the positive dataset, part of the data were collected by low-throughput literature mining in SmProt [29], i.e., they were biologically/ experimentally verified on the level of protein, cell, phenotype, etc. SmProt [29], which was released in 2016, is based on literature published by Dec 2015. We searched for new examples of verified micropeptides, supported by extensive experimental evidence, published after Dec 2015, and found 5 new micropeptides in the literature (Table 4). Among these 5 cases, 3 are actually already recorded in SmProt [34], however they were in the non-high-confidence subset, i.e., there was only indirect evidence on the presence of those micropeptides.

**Table 4:** List of micropeptides published after Dec 2015

Micropeptide name	Protein sequence length	in SmProt non-highConf	Reference
MOXI	56	yes	[42]
DWORF	35	yes	[43]
Myomixer / Minion	84	yes	[44]
SPAR	90	no	[45]
HOXB-AS3	53	no	[46]

in SmProt non-highConf: If this micropeptide was already included in the SmProt [34] non-high-confidence subset, then the value is “yes”, otherwise “no”

These 5 cases were taken as the **new_positive** dataset. They are analogous to “the future cases” if the time boundary were Dec 2015. One of the major purposes of MiPepid is for future prediction. Therefore, its performance on “future cases” matters.

We applied MiPepid on this new_positive dataset, and MiPepid correctly classified all of the 5 micropeptides. And this is another result showing the good generalization of MiPepid.

### Comparison with existing methods

#### Comparison with current ORF coding potential prediction methods

There are several state-of-the-art bioinformatics methods built to predict the coding/noncoding capability of a DNA sequence, including CPC [24], CPC2 [25], CPAT [26], CNIT [27], PhyloCSF [28], etc. However, all of them were designed to work on “average” transcript datasets, i.e., datasets that consist primarily of transcripts of regular-sized proteins and noncoding RNAs. In these methods, sORFs present in either an mRNA encoding a regular protein, or in a noncoding RNA, are generally penalized and are likely to be classified as noncoding; in the former case there is already a longer ORF present so shorter ones are treated as noncoding, and in the latter case the ORFs are automatically considered to be noncoding because they are found in “noncoding” RNAs. Therefore, despite the good performance of these methods in predicting regular-sized proteins, they may not be able to identify micropeptides, which also play critical biological roles.

In contrast, MiPepid is specifically designed to classify small ORFs in order to identify micropeptides. Here we chose CPC [24], CPC2 [25], and CPAT [26] as representatives of current methods and evaluated their performances on the hold-out blind test set as well as on the new_positive dataset, both of which the positive data are composed of high-confidence micropeptides.

As shown in table 5, while the 3 methods (CPC [24], CPC2 [25], CPAT [26]) performed exceptionally well on negative cases (100% accuracy), they indeed struggled to classify the positive cases.

**Table 5:** Comparison with existing methods on the blind test set and the new_positive dataset

Method	Blind test set						New_positive	
	Positive		Negative		Overall			
	#Correct	Accuracy	#Correct	Accuracy	*F*_1_	Accuracy	#Correct	Accuracy
CPC [24]	17	0.02	567	1.00	0.04	0.42	0	0.00
CPC2 [25]	61	0.07	567	1.00	0.14	0.45	0	0.00
CPAT [26]	261	0.32	567	1.00	0.48	0.60	3	0.60
MiPepid (our method)	789	0.96	542	0.96	0.96	0.96	5	1.00

positive: the positive subset of the blind test set;

negative: the negative subset of the blind test set;

overall: the overall performance on the blind test set;

#correct: the number of correctly classified cases by a method;

accuracy: #correct divided by the total number of cases in that dataset/subset;

*F*_1_: the *F*_1_score

The positive cases in the blind test set are sORFs of high-confidence micropeptides supported by at least 2 different types of experimental evidence. CPC [24] and CPC2 [25] considered over 90% of them as noncoding, while CPAT [26] did better with 32% accuracy but is still below half. In contrast, while MiPepid performed slightly worse on the negative cases (96%), it correctly classified 96% of the high confidence micropeptides. And regarding sORFs of the newly-published micropeptides, all of which are supported by protein-level and phenotypic evidence, CPC [24] and CPC2 [25] did not consider any of them to be coding, and CPAT [26] correctly classified only 3 out of 5. These results are not surprising as all three existing methods were trained on datasets primarily consisting of regular-sized proteins. It is clear from those results that sORFs are a special subpopulation of ORFs and predictions on which entail specially designed methods.

#### Comparison with sORFfinder

As mentioned in the Introduction section, sORFfinder predicts sORFs by calculating nucleotide frequency conditional probabilities of hexamers; however, the server is no longer accessible. We located a downloadable version at http://hanadb01.bio.kyutech.ac.jp/sORFfinder/ and ran it locally. sORFfinder does not provide a trained model for human sORFs, nor is there any human dataset included in this software. To conduct the comparison, we therefore used sORFfinder to train a model using our own training dataset and then evaluated on our test set. It took hours to train the model using sORFfinder, as compared to seconds needed for MiPepid.

As shown in table 6, sORFfinder correctly predicts around 87% of the examples in the test set, which is fairly good. However, it is clear that MiPepid performs significantly better. It is not surprising that sORFfinder achieved a similar performance to MiPepid. sORFfinder utilizes hexamer information and a naïve Bayes approach to calculate the posterior coding probability of a sORF given its hexamer composition. MiPepid uses 4-mer information, but rather than naïve Bayes, uses logistic regression to learn patterns from the data automatically. Notably, MiPepid achieves better classification using a much smaller feature vector, and much less computational time for training the model.

**Table 6:** Comparison with sORFfinder

Method	Blind test set					
	Positive		Negative		Overall	
	#Correct	Accuracy	#Correct	Accuracy	*F*1	Accuracy
sORFfinder	708	0.86	506	0.89	0.89	0.87
MiPepid (our method)	789	0.96	542	0.96	0.96	0.96

positive: the positive subset of the blind test set;

negative: the negative subset of the blind test set;

overall: the overall performance on the blind test set;

#correct: the number of correctly classified cases by a method;

accuracy: #correct divided by the total number of cases in that dataset/subset;

*F*_1_: the *F*_1_ score

## Discussion

### MiPepid’s predictions on non-high-confidence Mircopeptides

The SmProt database has a high-confidence subset, examples of micropeptides that are supported by multiple kinds of evidence; the rest of the data are non-high-confidence. We collected those data and obtained their corresponding DNA sequences using the same pipeline used for the positive dataset (see Methods). We then used MiPepid to predict the coding capabilities of those data. Overall, MiPepid predicted 74% of them as positive. Table 7 shows detailed results based on different data sources.

**Table 7:** MiPepid’s prediction on the non-high-confidence data in SmProt

Data source	#sORFs	avg sORF length (aa)	#Predicted positive	Proportion
high-throughput literature mining	25,663	44	20,516	0.80
ribosome profiling	13,715	36	8596	0.63
MS data	324	15	233	0.72

high-throughput literature mining: published sORFs that were identified using high-throughput experimental methods;

ribosome profiling: sORFs predicted from Ribo-Seq data;

MS data: sORFs predicted from MS data;

#sORFs: number of sORFs from a particular data source;

avg sORF length (aa): the average length of sORFs measured in number of amino acids;

#predicted positive: number of sORFs that are predicted as positive by MiPepid;

proportion: $$ \frac{\mathrm{avg}\ \mathrm{sORF}\ \mathrm{length}}{\#\mathrm{predicted}\ \mathrm{positive}} $$

As can be seen in table 7, among the over 25 k sORFs collected by high-throughput literature mining, MiPepid predicted 80% of them as positive, which is a fairly high proportion. There are only 324 sORFs derived from MS data, and MiPepid labeled 72% of them as positive. Note that, on average, MS sORFs are significantly shorter than those from other sources. In contrast, among the over 13 k Ribo-Seq derived sORFs, MiPepid only predicted 63% of them as positive. This is not very surprising as there has been debate on the reliability of predicting peptides from Ribo-Seq data; some investigators have argued that the capture of an RNA transcript by the ribosome does not always lead to translation [16], and that some of the ribosome associated RNAs found in Ribo-Seq may be regulatory or non-specifically associated.

We are interested in looking at the relationship between the length of a sORF and its coding probability predicted by MiPepid.

**Fig. 2:**
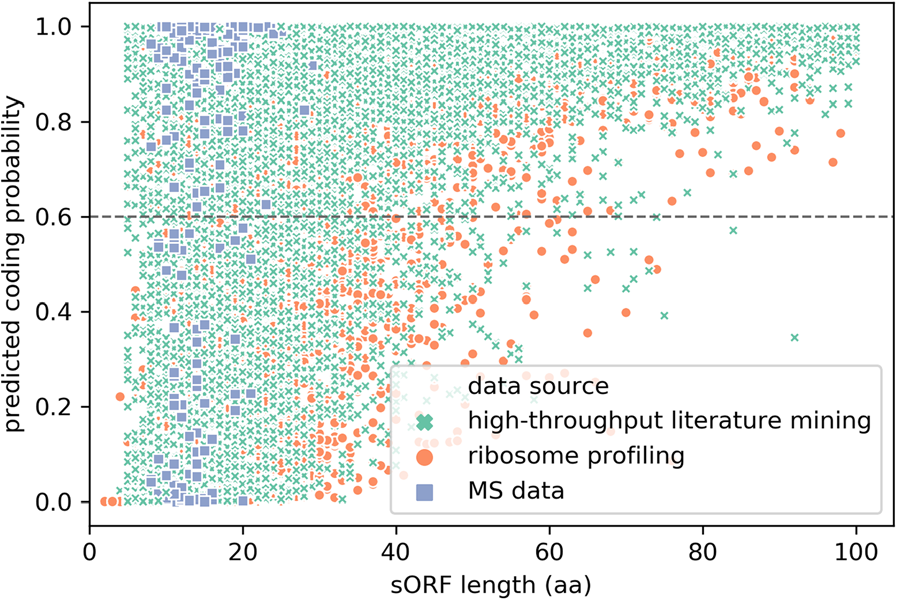
Predicted Coding Probability. Coding probability as a function of the predicted small ORF length. Scatterplot of the length of sORF vs. predicted coding probability for the non-high-confidence sORFs in SmProt. aa: number of amino acids. The *y* = 0.6 horizontal line separates sORFs that are predicted as positive (predicted coding probability ≥0.6) and the rest predicted negative

Figure 2 shows a moderately positive trend between the length of a sORF and its coding probability predicted by MiPepid. This is reasonable considering the following: (1) the longer a sORF, the less likely it occurs by chance; (2) the longer a sORF, the more 4-mer information it contains, which helps MiPepid to better classify it. Yet, we do see that for many very short sORFs (< 20 aa), MiPepid was able to identify the positives, and for long sORFs (> 50 aa), MiPepid was not misled by the length, and was still able to identify some as negatives. In Fig. 2, one can also see that sORFs derived from the MS data are very short (< 30 aa).

### MiPepid’s prediction on uORFs of protein-coding transcripts

A uORF (upstream open reading frame) is an ORF (usually short) located in the 5′-UTR (untranslated region) of a protein-coding transcript. A number of uORFs have been discovered to encode micropeptides and to play important roles in biological activities [47], and Ribo-Seq evidence suggests that many uORFs are translated [19]. uORFs have drawn increasing attention, and there is a great interest in determining the coding potentials of uORFs.

We extracted all possible small uORFs (from all 3 translation frames) of all annotated protein-coding transcripts in the Ensembl [34] human database. We then used MiPepid to determine the coding potentials of the extracted uORFs.

From 12,221 protein-coding transcripts, we extracted 42,589 small uORFs in total. 34.24% of the uORFs were predicted by MiPepid as coding. Among the 12,221 transcripts, 55.80% of them (6820) contain at least one potential micropeptide-encoding uORF. For the readers’ interest, we compiled all the small uORFs together with their coding potential score, location in the corresponding transcript, etc. into a Additional file 1. This file is available along with the MiPepid package.

### MiPepid’s prediction on lncRNAs

Long noncoding RNAs (lncRNAs) are RNA transcripts that lack a long ORF, and therefore were initially considered to be untranslated. Yet a growing number of lncRNAs have been discovered to be actually translated into functional micropeptides [36, 43, 45, 48].

We extracted all possible sORFs (from all 3 translation frames) of all human lncRNA transcripts in Ensembl [34] (those with the following biotypes: non_coding, 3prime_overlapping_ncRNA, antisense, lincRNA, retained_intron, sense_intronic, sense_overlapping, macro_lncRNA, or bidirectional_promoter_lncRNA). From the 26,711 lncRNA transcripts, we extracted 371,123 sORFs, averaging ~ 14 sORFs per transcript. 31.28% of the sORFs were predicted as coding. 86.63% of lncRNA transcripts were predicted to have at least one sORF that could potentially be translated into a micropeptide.

We present MiPepid’s prediction results on lncRNAs not for evaluating its performance but to show that the proportion of sORFs in lncRNAs that are “similar” to sORFs of high-confidence micropeptides in our training set is very high. It is impossible to evaluate MiPepid using the lncRNA results as we have very little data on which sORFs in lncRNAs are truly positive, and which are not. The results serve as a reference for researchers interested in further work on any of those lncRNAs. The Additional file 1 containing MiPepid results on the 26,711 annotated lncRNAs is also available in the MiPepid software package.

### MiPepid’s prediction on small protein-coding genes in other model organisms

MiPepid was trained on human data, and we expect that it would work well on related mammalian species, such as mouse, rat, etc. Yet, we want to know how well it generalizes to other species, e.g., plants, bacteria, etc. We therefore collected all annotated small protein-coding sequences (<= 303 bp) in *E. coli*, yeast, arabidopsis, zebrafish, and mouse from the Ensembl database [34], and examined whether they are predicted to be coding sequences by MiPepid. MiPepid sucessfully predicts at least 93% of the sequences as coding for these 5 species (Table 8). This indicates that MiPepid has been able to successfully learn generalized sequence patterns typical of human sORFs, and in addition, suggests that small protein-coding gene sequences share hidden patterns across biological kingdoms.

**Table 8:** MiPepid’s prediction on small protein-coding genes in model organisms

Species	#seq	%Predicted positive
*E. coli*	422	96.68%
yeast (*S. cerevisiae*)	502	93.63%
arabidopsis (*A. thaliana*)	2888	98.61%
zebrafish (*D. rerio*)	2481	96.78%
mouse (*M. musculus*)	6451	97.54%

#seq: number of small protein-coding sequences

%predicted positive: percentage of sequences predicted as coding by MiPepid

## Conclusions

MiPepid is designed to take a DNA sequence of a sORF and predict its micropeptide-coding capability. We suggest using sequences with transcriptome-level evidence, i.e., DNA sequences that are indeed transcribed, as MiPepid was trained to determine whether a transcript can be translated, and the training data did not include sORFs from untranslated DNA regions. The potential for an untranslated DNA sequence, such as an intergenic region, to be transcribed and translated was not addressed. MiPepid was specifically developed to predict small ORFs and “regular-sized” ORFs were not included in the training. Therefore, we recommend using MiPepid only on sORFs; MiPepid is not trained to efficiently predict long ORFs such as those found in typical mRNAs. MiPepid was trained on human data, but should work for related mammalian species, such as mouse, rat, etc. Retraining the model on other species requires only a set of known micropeptides and the corresponding genomic sequence.

## Availability and requirements

Project name: MiPepid

Project home page: https://github.com/MindAI/MiPepid

Operating system(s): Platform independent

Programming language: Python

Other requirements: Python 3, Numpy, Pandas, Pickle, Biopython

License: GNU GPL

Any restrictions to use by non-academics: None

## Additional file

Additional file 1: Supplemental Data - Tables 1-5. (XLSX 6674 kb)

## Data Availability

The MiPepid software and datasets are available at: https://github.com/MindAI/MiPepid.

## Abbreviations

5′-UTR: 5′ untranslated region
lncRNA: long noncoding RNA
miRNA: microRNA
ML: Machine learning
MS: Mass spectrometry
Ribo-Seq: Ribosome profiling
rRNA: ribosomal RNA
scaRNA: small Cajal body RNA
snoRNA: small nucleolar RNA
snRNA: small nuclear RNA
sORF / smORF: small open reading frame
tRNA: transfer RNA
uORF: upstream open reading frame
